# P-815. A Randomized Trial of Doxycycline vs. Trimethoprim Sulfamethoxazole (TMP-SMX) for Uncomplicated Skin and Skin Structure Infections (SSSIs)

**DOI:** 10.1093/ofid/ofaf695.1023

**Published:** 2026-01-11

**Authors:** Loren G Miller, Donna Phan Tran, Honghu Liu, Mary G Boyle, Evelyn A Flores, Kavitha Pathmarajah, Gregory J Moran, Mark Munekata, Linyu Zhou, Pluscedia Williams, Buchen (Olivia) Han, Leo Fukunaga, Stephanie A Fritz

**Affiliations:** Lundquist Institute at Harbor-UCLA Medical Center, Los Angeles, CA; Division of Infectious Diseases, the Lundquist Institute at Harbor-UCLA Medical Center, Torrance, CA, Torrance, California; University of California, Los Angeles, School of Dentistry, Public Health and Medicine, Los Angeles CA, Los Angeles, California; Washington University School of Medicine, St. Louis, Missouri; Division of Infectious Diseases, the Lundquist Institute at Harbor-UCLA Medical Center, Torrance, CA, Torrance, California; Olive View-UCLA Medical Center, Sylmar, CA; Olive View-UCLA Medical Center, Sylmar, CA; The Lundquist Research Institute at Harbor-UCLA Medical Center, Torrance, California; Public and Population Health, UCLA School of Dentistry, Los Angeles, California; Charles R. Drew University, Los Angeles, CA, Los Angeles, California; Lundquist Institute for Biomedical Innovation at Harbor-UCLA Medical Center, Torrance, California; Lundquist Institute for Biomedical Innovation at Harbor-UCLA Medical Center, Torrance, California; Washington University School of Medicine, St. Louis, Missouri

## Abstract

**Background:**

In the U.S., there are > 8 million visits for SSSIs annually, the vast majority of which are managed in the outpatient setting. However, safety and efficacy of doxycycline in comparison to other oral antibiotics for suppurative uncomplicated SSSIs (uSSSIs) remains unclear.

**Figure:**
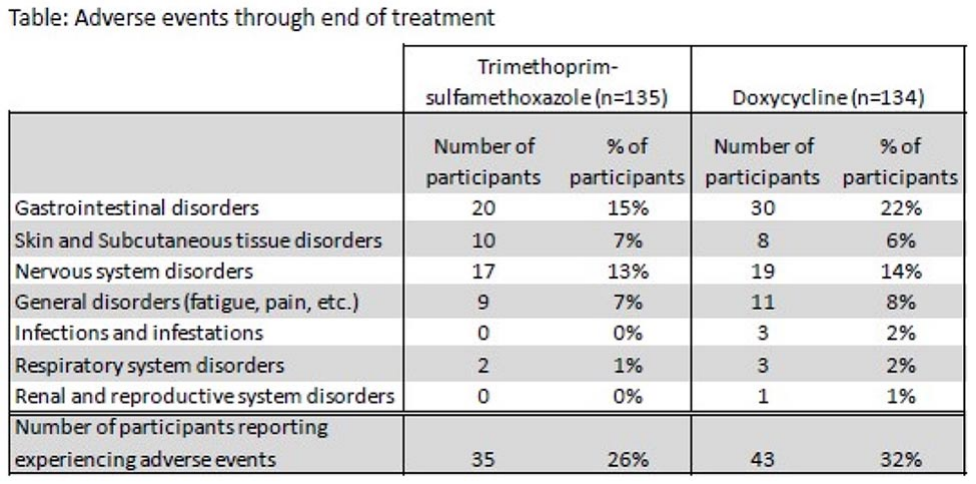

**Methods:**

We performed a double-blind, randomized controlled clinical trial of adults and children > 8 years of age with suppurative uSSSIs managed in the outpatient setting at 3 sites, two in California and one in Missouri. All participants were required to have adequate source control of uSSSI and were randomized to doxycycline 100 mg po or 1 double strength (DS) tablet of TMP-SMX twice daily. Doses of study medication were modified for children < 40 kg. Primary outcome was early clinical response (ECR) at 48-72 hours in the intent to treat population. Secondary outcomes included clinical response at end of treatment (EOT), clinical response at one month (1M) and adverse effects.

**Results:**

Among the 269 participants, 248 (92%) were adults and 21 (8%) were children; 132 (50%) were Hispanic, 93 (35%) African American, 33 (12%) white, 5 (2%) Asian, 1 (0.4%) Am. Indian/Alaskan Native, and 4 (1%) were other race/ethnicity. At the primary endpoint, ECR, cure rate was not significantly different between the two groups with a clinical response in 89/134 (66%) in the doxycycline arm in vs. 86/135 (64%) in the TMP-SMX arm (p=0.64). At the EOT visit, response was seen in 104 (78%) in the doxycycline arm and 104 (77%) in the TMP-SMX arm (p=0.91). Finally, at the 1M visit, clinical response was seen in 101 (75%) in the doxycycline arm in and 95 (70%) in the TMP-SMX arm (p=0.59). Adverse events were similar in the two groups with nausea (15 (11%) in the doxycycline arm vs. 10 (7%) in the TMP-SMX), headache (10 (7%) vs. 12 (9%)) and diarrhea (12 (9%) vs. 8 (6%)) being the most common. (Table)

**Conclusion:**

In our multi-center randomized trial of outpatient treatment of suppurative uSSSI performed in a diverse population, we found no significant differences in clinical response between doxycycline and TMP-SMX. Safety and tolerability were also similar between groups. For adults and children with suppurative uSSSIs undergoing source control, doxycycline appears to be a safe and efficacious alternative to TMP/SMX.

**Disclosures:**

Loren G. Miller, MD MPH, Armata: Grant/Research Support|GSK: Grant/Research Support|Merck: Grant/Research Support|Paratek: Grant/Research Support Gregory J. Moran, MD, FACEP, AbVacc: Grant/Research Support Mark Munekata, MD,MPH, Intuitive Surgical: Stocks/Bonds (Public Company)

